# Filling the gaps in *Leishmania naiffi* and *Leishmania guyanensis* genome plasticity

**DOI:** 10.1093/g3journal/jkab377

**Published:** 2021-11-13

**Authors:** Luz H Patiño, Marina Muñoz, Paula Pavia, Carlos Muskus, Maryia Shaban, Alberto Paniz-Mondolfi, Juan David Ramírez

**Affiliations:** 1 Facultad de Ciencias Naturales, Centro de Investigaciones en Microbiología y Biotecnología-UR (CIMBIUR), Universidad del Rosario, Bogotá, Colombia; 2 Hospital Militar Central, Bogotá, Colombia; 3 Programa de Estudios y Control de Enfermedades Tropicales (PECET), Universidad de Antioquia, Medellín, Colombia; 4 Incubadora Venezolana de la Ciencia/Instituto de Investigaciones Biomédicas IDB, Barquisimeto, Venezuela; 5 Department of Pathology, Molecular, and Cell-Based Medicine, Icahn School of Medicine at Mount Sinai, New York, NY 10029, USA

**Keywords:** *Leishmania naiffi*, *Leishmania*. *guyanensis*, copy number variation, phylogenetic analysis, single nucleotide polymorphisms

## Abstract

Insufficient and irregular data reports on Leishmaniasis, issuing from the developing world, have left much to be desired in terms of understanding the molecular signatures producing distinct infectious phenotypes of the disease. Herein, we report on the complete genome sequencing of *Leishmania naiffi* and *Leishmania guyanensis*, sampled from patients in regions of Colombia and Venezuela. In this study, the isolates of cutaneous lesions from both species presented limited structural variation at the chromosomal level, low gene copy number variation, and high genetic heterogeneity. We compared these sequences to the reference genomes hitherto related from Brazil and French Guyana. Although of the same species, we note a consequential genomic disparity between the Venezuelan and French Guyanese isolates of *L. guyanensis*. Although less significant on the global schema of cutaneous and mucosal disease, such genomic studies of *L. naiffi* and *L. guyanensis* substantiate the gaps in understanding of the molecular architecture and multivariate clinical pictures of Leishmaniasis, on an international scale.

## Introduction

Parasites of the genus *Leishmania* cause various diseases named leishmaniases; this parasite has been classified into four different subgenera: *Leishmania* (*Leishmania*), *Leishmania* (*Mundinia*), *Leishmania* (*Sauroleishmania*), and *Leishmania* (*Viannia*) (Espinosa *et al.* 2018), being *L.* (*Viannia*) and *Leishmania* (*Leishmania*) the subgenera with the highest distribution worldwide and with a genome size of ∼32 Mb (Butenko *et al.* 2019). Nine subgenera *L.* (*Viannia*) (sub)species have been described so far, including *L. naiffi* and *L. guyanensis* (Espinosa *et al.* 2018). Leishmaniasis is a parasite-effected disease state in mammals, which manifests in symptoms on a spectrum from tegumentary to visceral, depending on interplay between host, vector, and pathogen physiology. In South America, the cutaneous and mucocutaneous forms predominate, with Cutaneous Leishmaniasis (CL) and Mucocutaneous Leishmaniasis (MCL) being chiefly brought about by *Leishmania* *braziliensis*, *Leishmania* *panamensis*, and *L. guyanensis*. *Leishmania* *naiffi* has also been linked with CL cases (Ovalle-Bracho *et al.* 2019; Correa-Cardenas *et al.* 2020).

Since 1989, when the first clinical descriptions of CL by *L. naiffi* emerged from Brazil (Ducharme *et al.* 2020), DNA samples of this species have been retrieved from murine (Cassia-Pires *et al.* 2014; Roque and Jansen 2014) and sand fly hosts (Silva *et al.* 2021). Albeit rare to cause disease in humans (and in such cases, responsive to therapy), instances of CL are regularly described in connection to *L. naiffi* throughout South America (Correa-Cardenas *et al.* 2020; Ducharme *et al.* 2020; de Almeida *et al.* 2021). Some studies even denote parasite resistance to first-line therapy (Ducharme *et al.* 2020). In Colombia, *L. naiffi* infection has been reported in humans as well as in *Canis lupus familiaris* (Correa-Cardenas *et al.* 2020; Patiño *et al.* 2021).

*Leishmania* *guyanensis*, is contrarily a well-established instigator of CL and MCL in South America, being described throughout the continent, including regions of Colombia and Venezuela (Delgado *et al.* 1997; Couppie *et al.* 2004; Ovalle-Bracho *et al.* 2019; Olivier and Zamboni 2020; Santos *et al.* 2020). Infection by this species is likewise known to materialize in symptoms of Diffuse Cutaneous Leishmaniasis (DCL) and Disseminated Leishmaniasis (DL). To date, analyses of genomic singularities of *L. naiffi* and *L. guyanensis* from patient isolates in the endemic localities of Venezuela and Colombia have been sparse. Herein, we describe whole-genome sequencing of these two species, isolated from human cutaneous lesions of patients from Mérida, Venezuela, and Guaviare, Colombia.

## Materials and methods

### Sampling

This study accrued from two initial clinical samples of patients with CL: the S8104 isolated from a 51-year-old man in Merida, Venezuela and the HOMI-81 isolated from a 25-year-old male in Guaviare, Colombia.

### Genomic sequencing and assembly

Parasite isolates were obtained from samples of cutaneous lesions. The DNA was extracted and divided into two groups: the first, for species identification by nucleotide amplification and Sanger sequencing of the HSP70 gene; the second, for whole-genome sequencing following the protocols previously described (Patino *et al.* 2020). The paired-end Illumina reads obtained from the HOMI-81 and S8104 isolates were mapped to the reference genomes of *L. naiffi* LnCL223 and *L. guyanensis* LgCL085 (Coughlan *et al.* 2018) and assembled with the SMALT program (V-0.7.4; www.sanger.ac.UK/resources/software/smalt/). Statistics obtained during the sequencing of each isolate is presented in Supplementary Table S1.

### Evaluation of chromosome and gene copy number variations

For the chromosomal somy estimation, the median read depth of each chromosome was initially calculated (di). Subsequently, the median depth (dm) of the whole genome (35 chromosomes) for *L. guyanensis* and *L. naiffi* was calculated. Finally, the somy (*S*-value) of each chromosome was obtained using the following formula: *S* = 2 × di/dm (Cuypers et al. 2018). The ranges of somy (mono-di-tri-tetra and penta somy) were defined as previously described (Patiño *et al.* 2020). To evaluate the gene copy number variations (CNVs), we calculated and related the average haploid depth per gene without somy effect (dHG) and the full cell depth with somy effect (dFG) using the formula: (dFG = *S* × dHG). The statistical significance used in this study was set at a z-score cutoff of >2 and an adjusted *P*-value (Student’s *t*-test) of <0.05 (Patiño *et al.* 2020). The heatmaps were created using the *Heatmap3* package in R (Zhao *et al.* 2014).

### Interspecies phylogenetic inferences

Single nucleotide polymorphisms (SNPs) alignments from whole-nuclear and mitochondrial (maxicircle) genomes were used to evaluate the phylogenomic relationships between the isolates sequenced in this study, in addition to other species of the *L.* (*Viannia)* subgenus. Maximum likelihood trees were inferred using IQ-TREE 2 (Minh *et al.* 2020). The robustness of the nodes was evaluated using the Bootstrap (BT) method (with 1000 replicates). The obtained tree was visualized using the tool Interactive Tree Of Life V4 (http://itol.embl.de; Letunic and Bork 2019). To detect recombination signatures phylogenetic networks were built in SplitsTree5 (Huson and Bryant 2006) using the neighbor-net method. All metadata on genomes are summarized in Supplementary Table S2.

### SNPs analysis

Reads of each genome were mapped to corresponding reference genomes using the SMALT program (version 0.7.4) (http://www.sanger.ac.uk/science/tools/smalt-0). The Picard program (V-1.85) (http://broadinstitute.github.io/picard/) was used for merging and sorting bam files and marking duplicated reads, as described previously (Patiño *et al.* 2020). Additionally, the SNPs were called with the population-based Unified Genotyper method in the Genome Analysis Toolkit (GATK; version 3.4; https://software.broadinstitute.org/gatk/), where SNPs were called among all the samples simultaneously. Later, we realigned around indels to remove these and retrieved only the SNPs. GATK Variant Filtration was used to filter Low-quality SNPs, according to the following criteria: QD < 2.0 ‖ MQ < 40 ‖ FS > 60.0 ‖ ReadPosRankSum < −8.0. Once the SNPs were independently detected, the data were included in an Excel matrix, which was used to perform the comparative analysis. Finally, the data corresponding to the allele frequency (AF) were exported in txt files from the SNPs file using VCFtools recode option and SelectVariants—VariantsToTable options of GATK, and then, plotted using the plot function of R. The homozygous and heterozygous variant SNPs were determined from AF estimation data as reported elsewhere (Tihon *et al.* 2017).

## Results and discussion

### Nuclear and mitochondrial interspecies phylogenomic inferences

**Figure 1:**
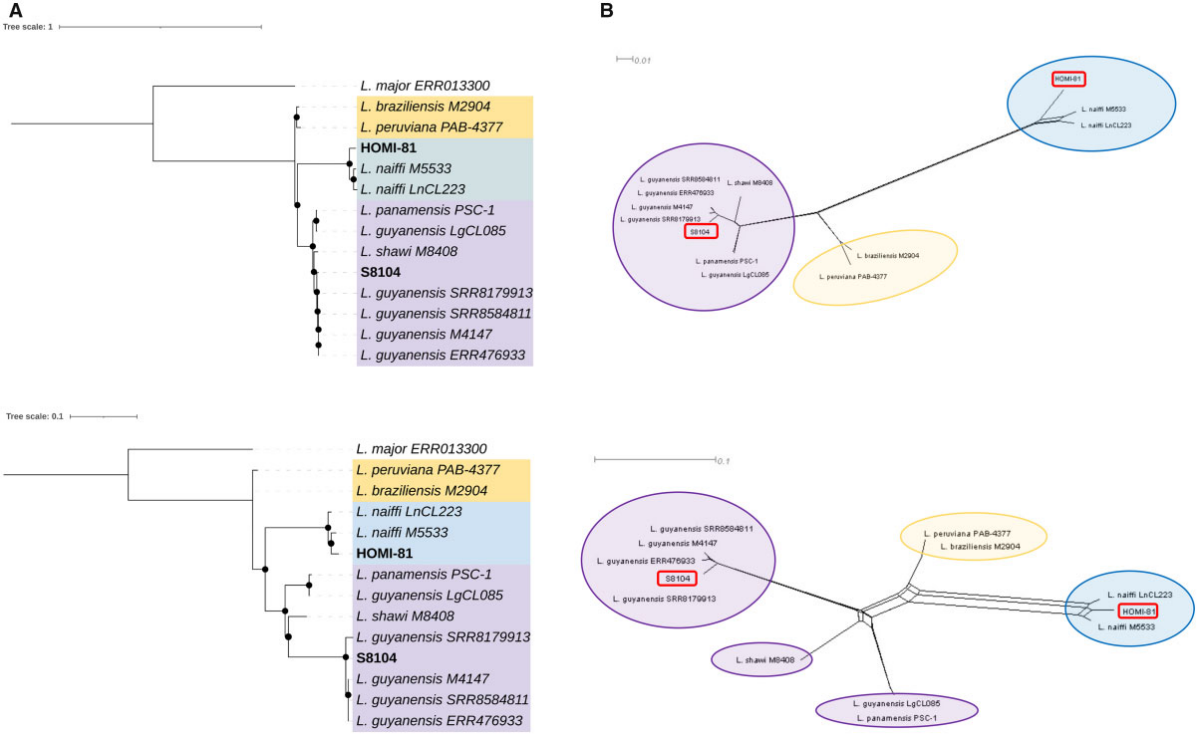
Nuclear and mitochondrial phylogenies of *L. naiffi* and *L. guyanensis* genomes analyzed. The trees located to the left of the figure represent the phylogenomic analysis based on nuclear (A) and mitochondrial: Maxicircle (B) SNP alignments of sequences analyzed in this study. *Leishmania naiffi LnCL223* and *L. guyanensis LgCL085* were used as reference genome of HOMI-81 and S8104 isolates, respectively and *Leishmania major ERR013300* was used as outgroup. Black dots represent well-supported nodes (BT ≥ 90%). The right panel represents the phylogenetic network (NeighborNet) constructed in SplitsTree 5, based on nuclear (C) and mitochondrial: Maxicircle (D) SNPs alignments for the genomes analyzed.

Two alignments were used to perform phylogenomic analyses. The first corresponded to SNPs from nuclear genome, where 345,167 variable sites and 264,298 parsimony-informative SNPs were identified. The second corresponded to SNPs from mitochondrial genomes where 156 variable sites and 128 parsimony-informative SNPs were identified. The results obtained demonstrate a close relationship between both Nuclear and Maxicircle SNPs of the HOMI-81 and S8104 genomes sequenced here, and the respective reference genomes of *L. naiffi* (*L. naiffi_LnCL223*) and *L. guyanensis* (*L. guyanensis LgCL085*; Coughlan *et al.* 2018; Figure  1, A and B). Furthermore, to analyze the genomes from *L. (Viannia)* subgenera, we noticed the formation of three independent clusters: cluster 1 (highlighted in light orange) included the genomes of *L. braziliensis* and *Leishmania* *peruviana*, the cluster 2 (highlighted in light blue), was represented for all *L. naiffi* genomes analyzed in this study and the cluster 3 (highlighted in light purple) included not only the *L. guyanensis* genomes herein analyzed but also the *L. panamensis* and *Leishmania* *shawi* genomes. These findings are supported by phylogenetic network topologies (Figure  1, C and D).

### Chromosomal and CNV

**Figure 2:**
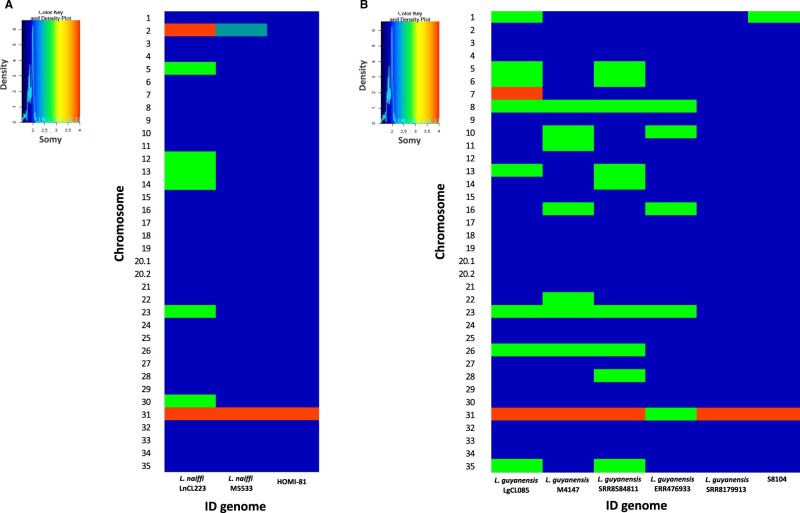
Evaluation of chromosomal copy number in the *L. naiffi* and *L. guyanensis* genomes analyzed. The heatmap shows the estimated copy number of the 35 chromosomes (*y*-axis) in the genomes analyzed (*x*-axis) (A) *L. naiffi* and (B) *L. guyanensis* genomes. Disomic (blue), trisomic (green), and tetrasomic (orange).

We noted chromosomal homogeneity across the genomes of HOMI-81 and S8104 isolates—their karyotypes being mostly disomic (Figure  2). We attribute the nominal genomic plasticity observed at the chromosomal levels of both isolates to be a likely effect of minimal recombination events, as had been previously demonstrated in *Trypanosoma brucei* subspecies (Almeida *et al.* 2018). Contrastively, low genomic plasticity could be due to recent introduction of these varieties to the latitudes in question, and the initial adaptation period of the species to novel human hosts, vectors, and zoonotic reservoirs.

We highlight structural similarities between the HOMI-81 isolate, and the Brazilian *L. naiffi* genome (M5533) of Edentata/*Dasypus* origin (Figure  2A). This line of evidence implies that the *L. naiffi* strain localized to Colombia could be derivative of a sylvatic variety adapted to continuous displacement within regions where zoonotic reservoirs and sand fly vectors are abundant (Kato *et al.* 2013; Ferro *et al.* 2015). Such reasoning has important epidemiological implications in Colombia as political unrest occasions continuous human displacement in the country of people between rural and urban areas. A similar scenario applies to Venezuela, a country facing a serious humanitarian crisis, which has led to massive displacement of refugees and migrants to neighboring countries. By default, human migration equates to dispersion of parasite species.

In addition, we ascertained greater structural similarity between the S8104 isolate, and the genome from French Guyana (*L. guyanensis*_SRR8179913), than the four Brazilian genomes analyzed in this study (*L. guyanensis* LgCL085, *L. guyanensis* M4147, *L. guyanensis* SRR8584811, and *L. guyanensis* ERR476933; Figure  2B). Calibrating these findings with the geographical proximity of these genomes, and the inherent capacity of adaptation/evolution of *L. guyanensis* to diverse ecological niches, we surmise that the pathogenic *L. guyanensis* strain circulating in Brazil is structurally dissimilar to the strains of the same species, issuing from the Caribbean coast of South America. Such suppositions cannot however be confirmed until more whole-genome studies of *L. guyanensis* are carried out, alike to this one.

Moreover, we observed low frequency of CNV amongst the genes on both isolates (82 genes in HOM1-81 and 66 genes in S8104). A total of 71 and 40 CNV genes were shared between all *L. naiffi* and *L. guyanensis* genomes analyzed respectively (Supplementary Tables S3 and S4). Interestingly, these genes were associated with survival, virulence; drug/ROS stress resistance, host immune evasion, glucose metabolism, and metastasis. Additionally, we highlight the genes that encode telomere-associated mobile elements DNA, which until date, have only been described in *L. braziliensis*, *L. panamensis*, *L. guyanensis*, and *L. naiffi* (Coughlan *et al.* 2018) and the genes associated with autophagy (ATG8/AUT7/APG8/PAZ2), as showing high CNV in *L. guyanensis* but not in *L. naiffi* species.

### SNP analysis

In the terms of SNPs we report higher genetic heterogeneity in the HOMI-81 isolates (214,474 SNPs) as compared with 85,238 SNPs within *L. naiffi* M5533 isolates (Supplementary Table S5). Such distinct characters of genetic variability between the two strains could be the result of their historical geographic distributions and the coordinate exigency to have adapted to the ecological environments of everchanging hosts. More proficient sampling of *L. naiffi* is necessary to confirm these hypotheses.

Regarding the S8104 isolates, we identified 131,626 SNPs within the *L. guyanensis* genome—agreeing with the equally high heterogeneity of the remaining *L. guyanensis* genomes analyzed (Supplementary Table S5). Thereby, our findings confirm that *L. guyanensis*, like *L. braziliensis* (Patiño *et al.* 2020) present a high degree of genetic variability. As in the case of *L. naiffi*, this variability is to be associated with the equally diverse opportunities had by the parasite to install itself in variable arthropod vector species and zoonotic hosts (Rotureau *et al.* 2006; Ramirez *et al.* 2016). The genetic heterogeneity of *L. guyanensis* could parallel its capacities to instigate diverse clinical pictures of disease and resistance to common therapies (Borges *et al.* 2018). Although previous studies describe that *L. guyanensis*, *L. panamensis*, and *L. shawi* are a monophyletic species complex as demonstrated through Multilocus Sequence Analysis (MLSA), Multilocus Enzyme Electrophoresis (MLEE), and hsp70 analysis (Coughlan *et al.* 2018), the whole-genome analysis of these species, has allowed to evidence a considerable genomic variability between them, in terms of SNPs/indels and gene and chromosome CNVs (Coughlan *et al.* 2018), which could support the idea that although they are closely related, they could be distinct species, as was demonstrated when comparing the whole genome of *L. braziliensis* and *L. peruviana* (Valdivia *et al.* 2015). However, we believe that the analysis of a larger number of genomes is necessary to clarify if these are distinct species or belong to a single genetic group.

In conclusion, this is the first study to report the whole-genome sequence of Colombian *L. naiffi* and Venezuelan *L. guyanensis* isolates. A detailed genomic analysis of both isolates has demonstrated similar low structural variability at the chromosomal level, across the board. We observed high genetic heterogeneity on the basis of generous SNPs, which we attribute to the initial adaptation process of the species to new human hosts and novel environmental niches, not necessarily involving genetic alterations at the structural level. Nevertheless, new isolates need to be sequenced to support these hypotheses. 

## Data availability

The dataset generated during the study was deposited at DDBJ/ENA/GenBank under the study accession number PRJEB46091.

Supplementary material is available at *G3* online.

## Supplementary Material

jkab377_Supplementary_Data
